# Identification and characterization of novel NF-kB dependent genes involved in HTLV-I pathogenesis

**DOI:** 10.1186/1742-4690-12-S1-P93

**Published:** 2015-08-28

**Authors:** Zhaoxia Qu, Gutian Xiao

**Affiliations:** 1 University of Pittsburgh Cancer Institute, Department of Microbiology and Molecular Genetics, University of Pittsburgh School of Medicine, Pittsburgh, PA, USA

## 

The NF-kB transcription factor plays pivotal roles in the pathogenesis and therapy-resistance of human cancers, including adult T-cell leukemia (ATL) induced by the oncoretrovirus HTLV-I. However, the downstream target genes of NF-kB involved in cancer biology and therapy remain largely unknown. To address this important issue, we have developed a novel approach called subtraction-based complementary gene expression cloning strategy. Given the characteristic anti-apoptosis activity of cancer cells, we used this approach to identify NF-kB-dependent anti-apoptotic genes involved in HTLV-I oncogenesis. The principle of this strategy is that expression of anti-apoptotic genes induced by HTLV-I-activated NF-kB should protect normal T cells from apoptosis induced by death inducers such as FasL. Briefly, a subtractive cDNA retroviral library enriched in genes induced by HTLV-I-NF-kB was generated and used to infect FasL-sensitive T cells. The infected T cells were treated with FasL and G418 (selective marker of cDNA expression). The FasL-and G418-resistant clones were isolated by limiting dilution, and the functional genes involved in FasL-resistance were fished out by RT-PCR and DNA sequencing. Using this strategy, several known NF-kB-dependent apoptotic genes have been identified, such as IAP1, Bcl-xL, c-FLIP and DcR2, indicating the reliability of our approach. Notably, numerous novel NF-kB-dependent anti-apoptotic genes were also identified. One of these novel genes has been confirmed to be expressed highly in HTLV-I-transformed T cells and primary ATL cells, and can be induced in normal T cells by HTLV-I in an NF-kB-dependent manner. Our mechanistic studies further indicate that this novel protein binds to mitochondria and prevents FasL activation of Bid, Caspase 9 and Caspase 3 but not Caspase 8. Currently, we are actively investigating the pathophysiological role of this novel gene in the biology and therapy of ATL and other cancers associated with deregulated NF-kB.

